# Effectiveness and Safety of Intralesional Dutasteride in Patients With Androgenic Alopecia: A Systematic Review and Meta‐Analysis

**DOI:** 10.1111/jocd.70560

**Published:** 2025-12-10

**Authors:** Abdullah Almeziny, Asail Alghamdi, Alhanoof Alajlan, Norah A. Almarhab, Areen E. Almatham, Esraa A. Shaheen, Saad Altalhab, Abdullah Ibrahim Alkhalifah

**Affiliations:** ^1^ College of Medicine Imam Mohammad Ibn Saud Islamic University Riyadh Saudi Arabia; ^2^ Dermatology Department King Fahad Hospital Albaha Saudi Arabia; ^3^ College of Medicine King Saud University Riyadh Saudi Arabia; ^4^ College of Medicine Umm Al‐Qura University Al‐Qunfudhah Saudi Arabia; ^5^ Qassim University Buraydah Saudi Arabia; ^6^ King Salman Medical City Medinah Saudi Arabia; ^7^ Department of Dermatology, College of Medicine Imam Mohammad Ibn Saud Islamic University Riyadh Saudi Arabia; ^8^ Prince Sultan Military Medical City Riyadh Saudi Arabia

**Keywords:** androgenic alopecia, efficacy, intralesional dutasteride, safety, systematic review

## Abstract

**Background:**

Androgenic alopecia (AGA) is a common condition characterized by progressive hair loss influenced by dihydrotestosterone (DHT). While oral dutasteride has shown efficacy in treating AGA, concerns about systemic side effects have prompted interest in localized treatments such as intralesional administration.

**Aims:**

This systematic review evaluates the effectiveness and safety of intralesional dutasteride for treating AGA in adults.

**Methods:**

Following PRISMA guidelines, we conducted a comprehensive search of multiple databases for studies involving adults (≥ 18 years) with AGA treated with intralesional dutasteride. Eligible studies included randomized controlled trials, non‐randomized studies, cohort studies, and observational studies. Exclusion criteria were animal studies, duplicate publications, and studies lacking relevant outcome data. The primary outcomes were improvements in hair density, hair thickness, and photographic evidence of hair growth. Secondary outcomes assessed safety, including local and systemic adverse events. Data extraction and quality assessment were independently performed by two reviewers.

**Results:**

Included studies consistently reported improvements in hair density and thickness following intralesional dutasteride treatment, with photographic evidence supporting visual improvement. Treatment was generally well tolerated, with minimal adverse events such as mild scalp irritation and no significant systemic effects reported. However, the studies varied in methodology, sample size, and follow‐up duration.

**Conclusions:**

Intralesional dutasteride appears to be a promising treatment option for AGA, offering localized efficacy with a favorable safety profile. Nonetheless, the current evidence is limited by heterogeneity and a lack of large‐scale, high‐quality trials. Further standardized research is needed to confirm these findings.

## 1 Introduction

Androgenetic alopecia (AGA) is the most frequent form of hair loss, affecting both men and women [1]. By the age of 70, signs of AGA are present in 80% of Caucasian men and up to 40% of women [2, 3]. It is a multifactorial, polygenetic condition influenced by factors such as endocrine imbalances, circulating androgens, and microinflammation [4, 5, 6]. AGA often has a significant impact on quality of life, frequently causing psychological distress [7, 8]. The underlying pathological mechanism primarily involves the conversion of testosterone to dihydrotestosterone (DHT) via the enzyme 5‐α‐reductase (5αR) [9].

Treating androgenic alopecia (AGA) presents a challenge due to its complex underlying mechanisms, with the primary goals being to stop its progression and prevent further hair thinning. Currently, there are only two FDA‐approved treatments for AGA: minoxidil, a vasodilator that may lead to unwanted hair growth and finasteride, a type II 5‐alpha reductase inhibitor known for its potential sexual side effects [10]. Dutasteride, a second‐generation 5‐alpha reductase inhibitor, is considered more effective than finasteride because it inhibits both type 1 and type 2 forms of the enzyme, leading to a 90% reduction in DHT serum levels, compared to the 70% reduction achieved by finasteride [11]. Early studies comparing finasteride and dutasteride emerged in the 2000s [12, 13, 14].

Due lack of a similar review for the role of intralesional dutasteride in Patients with Androgenic Alopecia, the current review aimed to assess the effectiveness and safety of intralesional dutasteride in Patients with Androgenic Alopecia. This will lead to evidence regarding intralesional dutasteride, ultimately leading to informed conclusions and recommendations.

## 2 Methodology

This systematic review aims to assess the effectiveness and safety of intralesional dutasteride for treating androgenic alopecia (AGA). By following the PRISMA guidelines, this review provided a clear, structured approach to synthesizing the available evidence, offering insights into this treatment's benefits and potential risks. The authors focused on adults (≥ 18 years) diagnosed with androgenic alopecia which is characterized by a progressive thinning of hair and is influenced by genetic, hormonal, and environmental factors. The study focuses on using intralesional dutasteride as a treatment option for this condition, examining its effectiveness in promoting hair regrowth and assessing its safety profile, regardless of the severity or duration of the condition. Individuals with hair loss conditions other than androgenic alopecia (e.g., alopecia areata, telogen effluvium, scarring alopecia), those on participants who have used other hair loss treatments (e.g., topical minoxidil, oral finasteride, or corticosteroids) within a specified timeframe before the study (e.g., 3–6 months), and pregnant or nursing women, due to potential risks associated with dutasteride were excluded. Only studies that evaluate intralesional dutasteride were considered, and they were compared to placebo, no treatment, or other treatments such as oral dutasteride or minoxidil. The main outcomes of interest were the effectiveness of the treatment, as measured by hair density, hair thickness, or photographic evidence of hair growth. Secondary outcomes included safety aspects, specifically the frequency and types of adverse events. Eligible studies will include randomized controlled trials (RCTs), non‐randomized clinical trials, cohort studies, and observational studies. Studies that lack relevant data or involve animals or non‐human participants were excluded. The literature search was thorough, covering databases like PubMed, Scopus, Embase, Web of Science, and the Cochrane Library, as well as gray literature and trial registries like ClinicalTrials.gov. Keywords included terms related to androgenic alopecia and intralesional dutasteride, ensuring we capture a broad spectrum of studies. Two independent reviewers screened the titles and abstracts to determine eligibility based on predefined criteria, and any disagreements were resolved through discussion or with the involvement of a third reviewer. Full‐text versions of the studies will then be assessed for final inclusion. Data extraction focused on study characteristics, such as author, year, country, and study design, as well as demographic information (e.g., age, gender, AGA severity), intervention details (dose, frequency, and duration of treatment), and outcomes (effectiveness and safety). Two independent reviewers extracted the data, ensuring consistency, and resolving any discrepancies through discussion or with the help of a third reviewer.

### 2.1 Statistical Analysis

R markdown document (v2.29) at RStudio (v2024.09.1) with R (v4.4.2) was used to perform a meta‐analysis of the selected studies. The prevalence with 95% CI was used to assess the improvement rate, total adverse effects, pain and frontal edema complications after Dutasteride. Additionally, the mean difference with 95% CI was used to compare the change in terminal hair count, vellus hair count and hair density after Dutasteride. The random effects model and inverse variance method were used in order to reduce heterogeneity. A “dmetar” package for leave‐one‐out meta‐analysis and its plot was utilized to solve the heterogeneity. Additionally, subgroup analyses based on the duration of treatment and drug regimen were done whenever possible. In terms of values, we interpreted the I‐square as follows: not significant for 0%–40%, moderate heterogeneity for 30%–60%, and substantial heterogeneity for 50%–90%, following the Cochrane Handbook chapter nine. Publication bias was assessed using a funnel plot.

## 3 Results

### 3.1 Study Characteristics

A total of eight studies, were included in this systematic review and meta‐analysis. Study designs included two prospective cohort studies [15, 16], two retrospective studies [17, 18]. In addition, there were three randomized controlled trials [19, 20, 21], and one case series [22]. Sample sizes ranged from 1 to 541, with a mix of males and females across studies. The median age of participants in [17] was 47 years (range 15–83), while mean ages in other studies varied, such as 41.2 years [18]. Study durations ranged from 3 months [15, 16, 23] to a median of 17 months [17]. Check Table 1.

**TABLE 1:** Characteristics of the included studies.

Authors	Study design	Intervention	Sample size	Inclusion	Results/conclusion
Saceda Corralo et al. (2022) [17]	Retrospective study	Mesotherapy with lidocaine, minoxidil, or dutasteride	541	Patients who were treated and had at least 6 months of follow‐up.	After 1 year, 86 patients (15.9%) who had hair loss responded well to dutasteride mesotherapy, with the majority exhibiting clinical improvement. The most frequent adverse effect was pain, and for certain people, it might be a good alternative
Melo et al. (2022) [18]	Multicenter retrospective, descriptive study	Mesotherapy	14 patients	14 instances of frontal edema brought on by mesotherapy for the treatment of AGA	After mesotherapy for AGA, the study finds frontal edema, which often appears in the first two sessions and lasts for 1–4 days. Lidocaine was utilized as the anesthetic, while dutasteride and minoxidil were possible contributing factors. Dermatologists need to be aware of this possible adverse impact
Sobhy et al. (2013) [19]	Randomized controlled trial	Oral minoxidil (1–5 mg daily) and dutasteride micro injections (0.01%)	90 male patients	The person is between the ages of 18 and 55, has normal baseline semen and serum DHT levels, is not pursuing pregnancy, and has no serious medical issues.	The best mesotherapy for male pattern hair loss is a solution containing dutasteride, which reduces or stops hair loss while encouraging the growth of new hair
El‐Komy et al. (2017) [24]	Case series	Intradermal injection of Dutasteride	3 female	Three patients with androgenetic alopecia who had started to lose their hair, after receiving irregular mesotherapy sessions for a year.	In order to guarantee a safe and efficient course of treatment, mesotherapy for androgenetic alopecia must be properly regulated and monitored because it may result in hair loss and scarring
Moftah et al. (2013) [16]	Prospective cohort study	Tiny needle placement Minoxidil and dutastride together made up Group A, whereas dutastride made up Group B.	51 female patients (40 patients were included in the analysis)	FPHL patients	Mesotherapy for androgenetic alopecia may result in hair loss and scarring, according to the study, underscoring the necessity of appropriate regulation and oversight of its usage
Essam et al. (2024) [15]	Prospective comparative study	A single milliliter of intradermal injections of dutasteride 0.01% (Dutasteride 0.01%, Mesotherapy Worldwide, Australia)	40 female	FPHL patients	Despite non‐significant changes in trichoscopic findings before and after treatment, the study indicated that treating FPHL with a combination of minoxidil and dutasteride treatments following scalp microneedling was more effective than using dutasteride alone
Abdallah et al. (2008) [20]	Single blinded, placebo‐controlled study	Mesotherapy with dutasteride, variable doses	34 males	Twenty‐eight male with MPHL types III, IV, and V patients.	For moderate cases of MPHL, mesotherapy with a solution containing dutasteride works well and has few adverse effects. It is advised that more research be done using dutasteride alone and for longer durations
Moftah et al. (2013) [16]	Placebo controlled study.	Group I injected with dutasteride 0.5 mg, biotin 20 mg, pyridoxin 200 mg and D‐panthenol 500 mg. group II injected with 0.9% saline.	126 Female	FPHL patients	Monotherapy with a preparation containing dutasteride improved hair diameter and reduced epilated hairs in FPHL patients by 62.8%, whereas the control group saw improvements of 17.5%. There were few adverse effects, and there was no significant correlation between the improvement and the length of the illness

Abbreviations: AGA, androgenetic alopecia; BASP, basic and specific classification; DHT, dihydrotestosterone; FPHL, female pattern hair loss; MPHL, male pattern hair loss.

### 3.2 Outcomes

#### 3.2.1 Terminal Hair Count

##### 3.2.1.1 Primary Analysis

**FIGURE 1:**
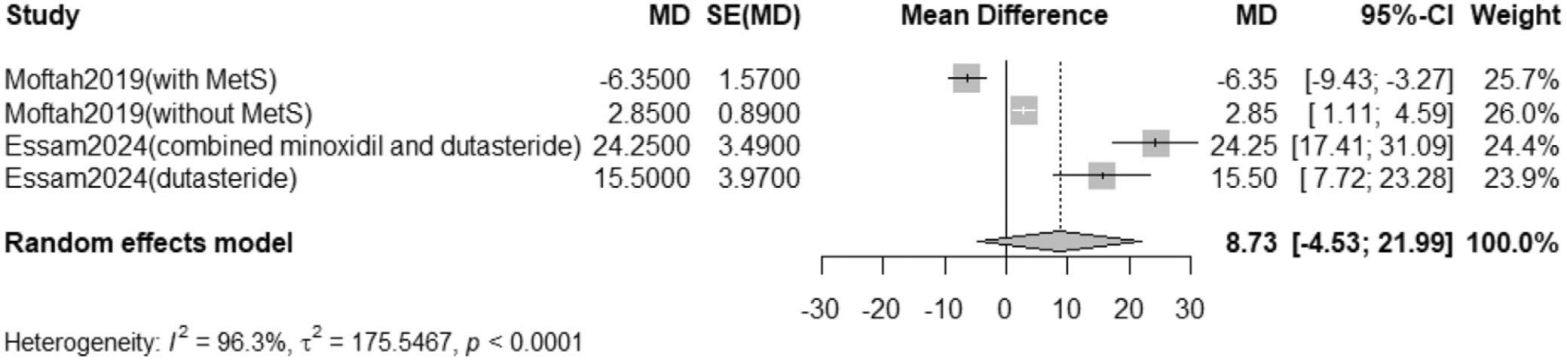
Forest plot of terminal hair count outcome.

**FIGURE 2:**
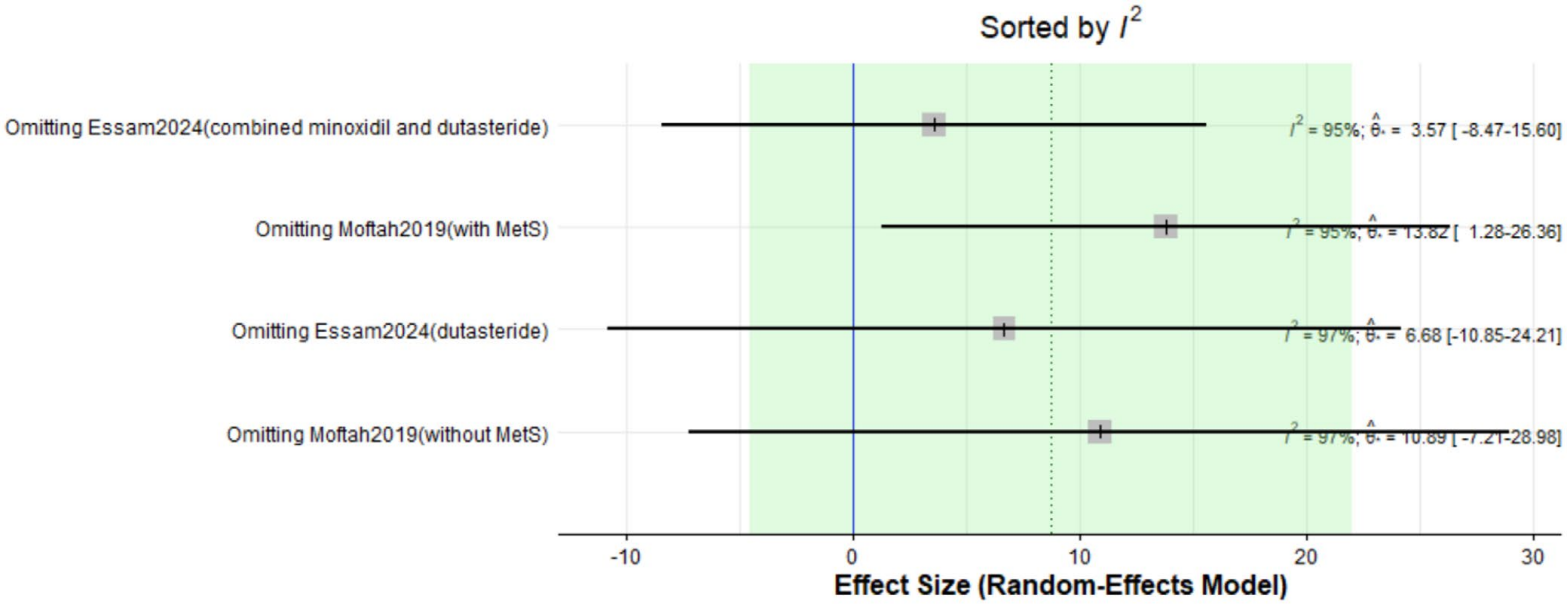
Sensitivity test of terminal hair count outcome.

**FIGURE 3:**
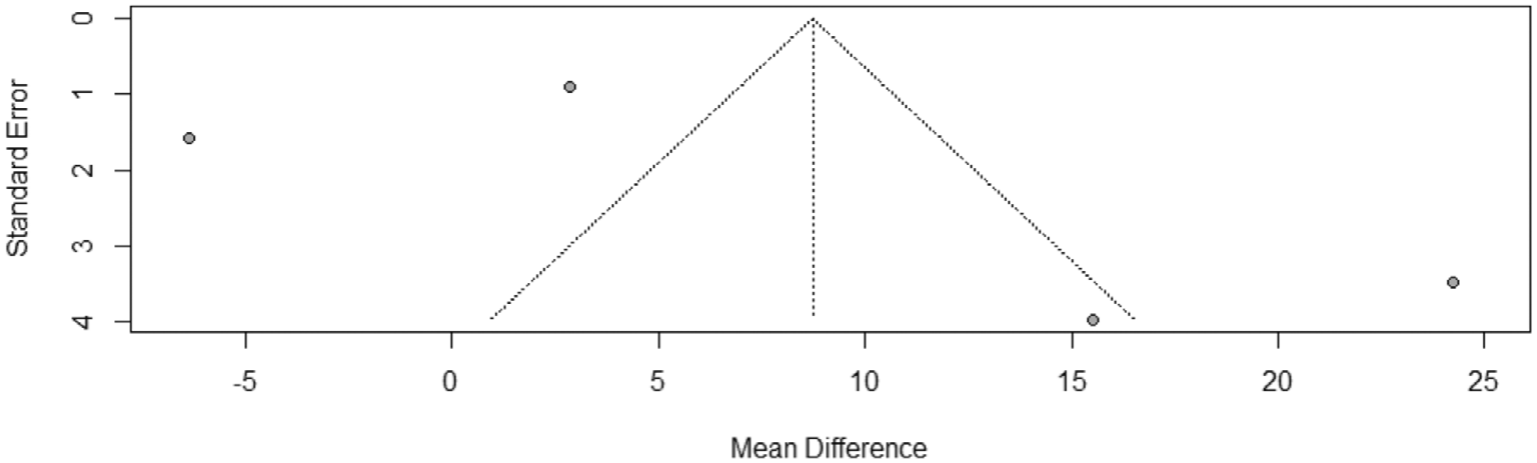
Funnel plot of terminal hair count outcome.

The pooled mean difference of the change in terminal hair count after Dutasteride was 8.73 with a 95% confidence interval (CI) ranging from −4.53 to 21.99 (Figure 1). The result was statistically significant (*p* < 0.0001). The sensitivity analysis revealed substantial heterogeneity among our included (*I* ^2^ = 96.3%). The leave‐one‐out test did not reduce the heterogeneity (Figure 2). The funnel plot of the change in terminal hair count is shown in (Figure 3).

##### 3.2.1.2 Drug Subgrouping

**FIGURE 4:**
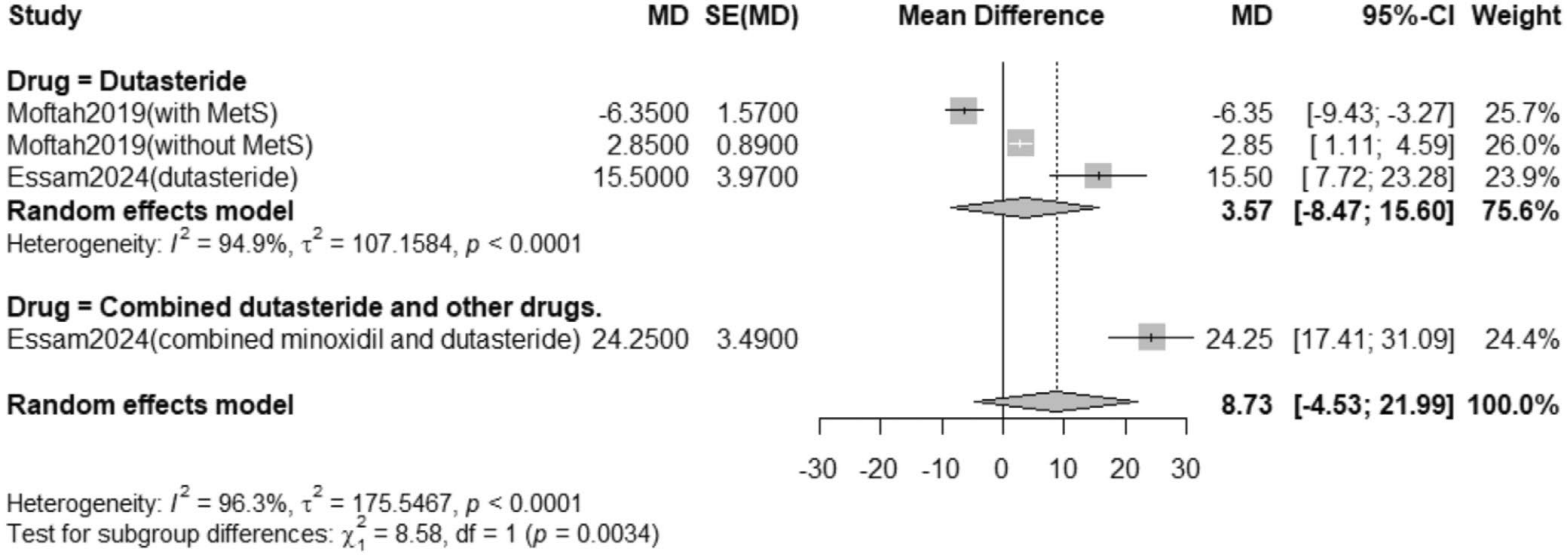
Subgrouping of the change in terminal hair count outcome.

The subgroup analysis revealed that the pooled mean difference of the change in terminal hair count after Dutasteride only was 3.57 with a 95% confidence interval (CI) ranging from −8.47 to 15.60 with a statistically significant (*p* < 0.0001) result (Figure 4).

#### 3.2.2 Vellus Hair

##### 3.2.2.1 Primary Analysis

**FIGURE 5:**
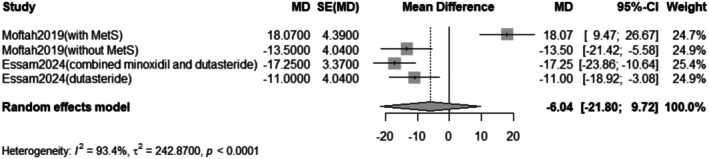
Forest plot of vellus hair count outcome.

**FIGURE 6:**
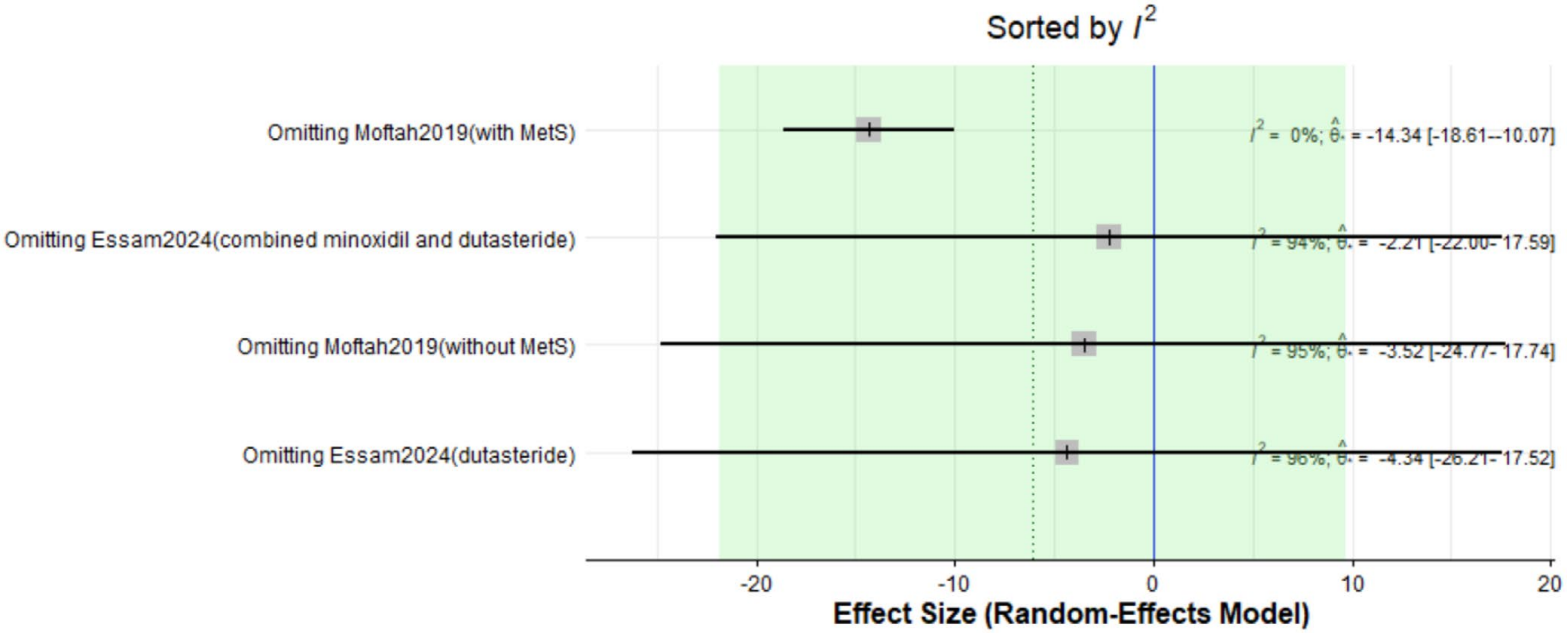
Sensitivity test of vellus hair count outcome.

**FIGURE 7:**
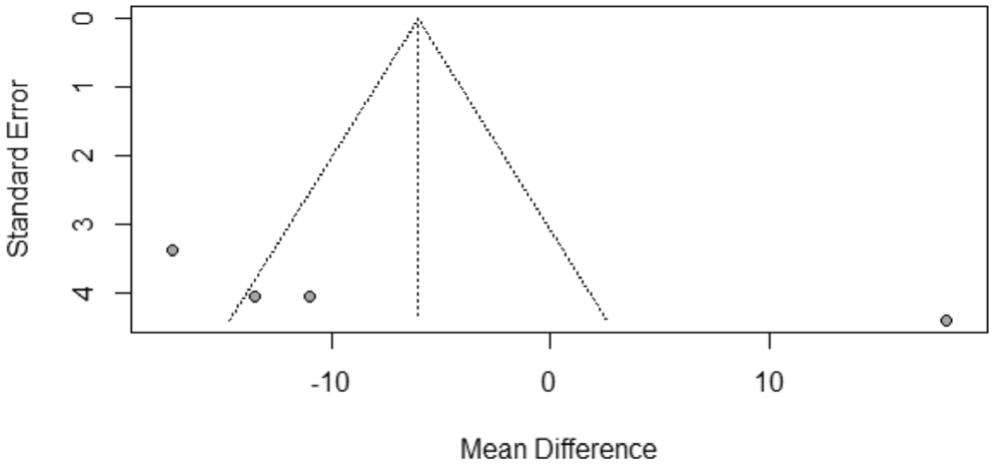
Funnel plot of vellus hair count outcome.

The pooled mean difference of the change in vellus hair count after Dutasteride was −6.04 with a 95% confidence interval (CI) ranging from −21.80 to 9.72 (Figure 5). The result was statistically significant (*p* < 0.0001). The sensitivity analysis revealed substantial heterogeneity among our included studies (*I* ^2^ = 93.4%). The leave‐one‐out test resolved the heterogeneity after omitting Moftah [16] (with Mets) (Figure 6). The funnel plot of the change in vellus hair count is shown in (Figure 7).

##### 3.2.2.2 Drug Subgrouping

**FIGURE 8:**
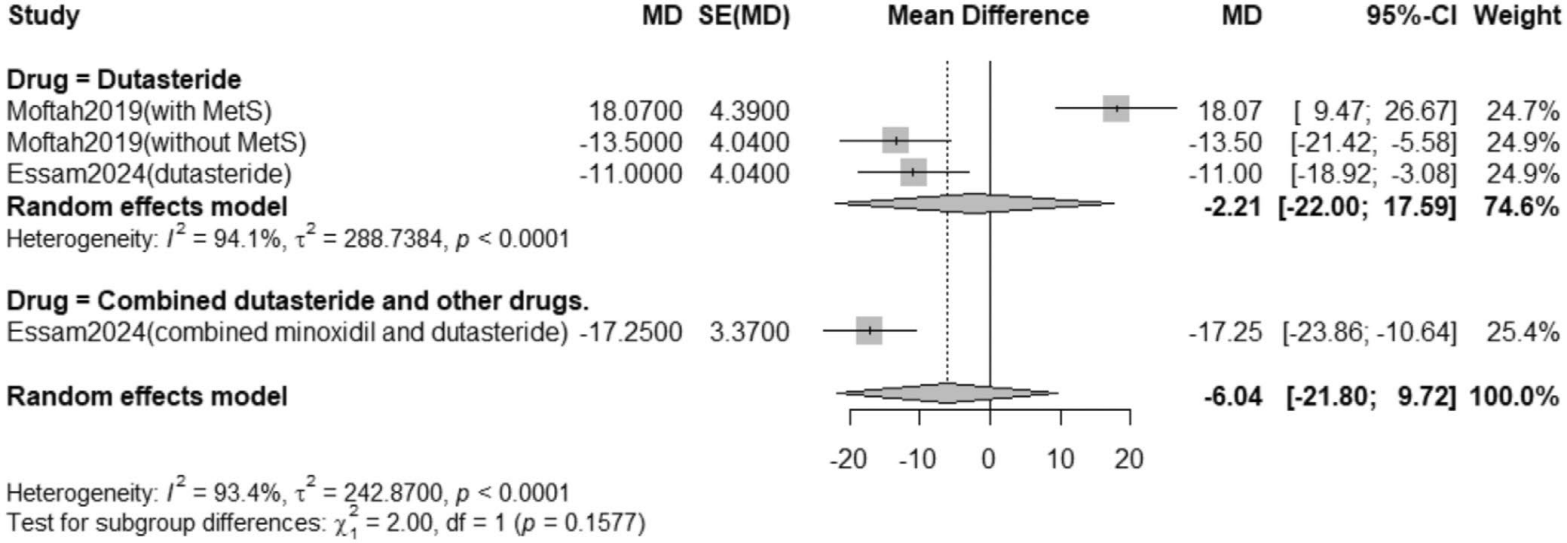
Subgrouping of the change in vellus hair count outcome.

The subgroup analysis revealed that the pooled mean difference of the change in vellus hair count after Dutasteride only was 3.57 with a 95% confidence interval (CI) ranging from −8.47 to 15.60 with a statistically significant (*p* < 0.0001) result (Figure 8).

#### 3.2.3 Hair Density

**FIGURE 9:**
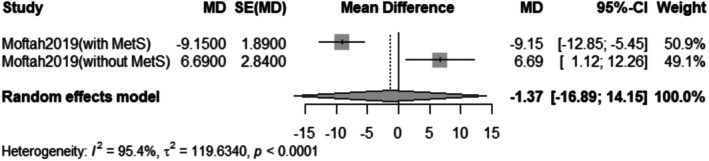
Forest plot of hair density outcome.

**FIGURE 10:**
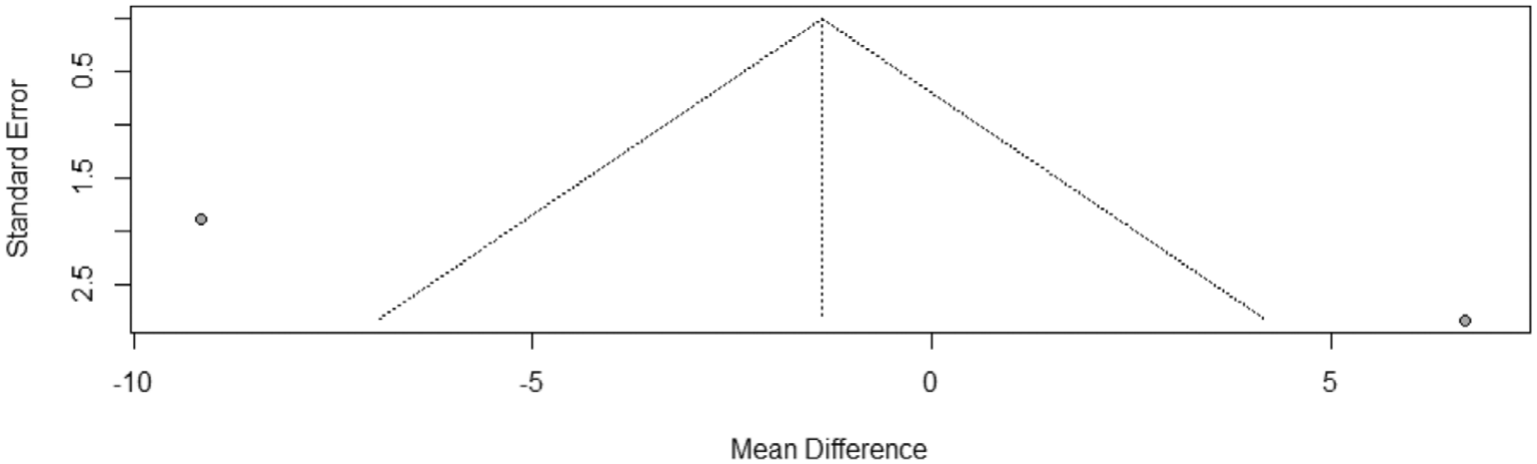
Funnel plot of hair density outcome.

The pooled mean difference of the change in hair density after Dutasteride was −1.37 with a 95% confidence interval (CI) ranging from −16.89 to 14.15 (Figure 9). The result was statistically significant (*p* < 0.0001). The funnel plot of the change in hair density is shown in (Figure 10).

#### 3.2.4 Improvement Rate

##### 3.2.4.1 Primary Analysis

**FIGURE 11:**
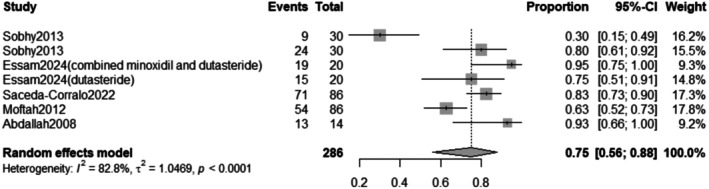
Forest plot of improvement rate outcome.

**FIGURE 12:**
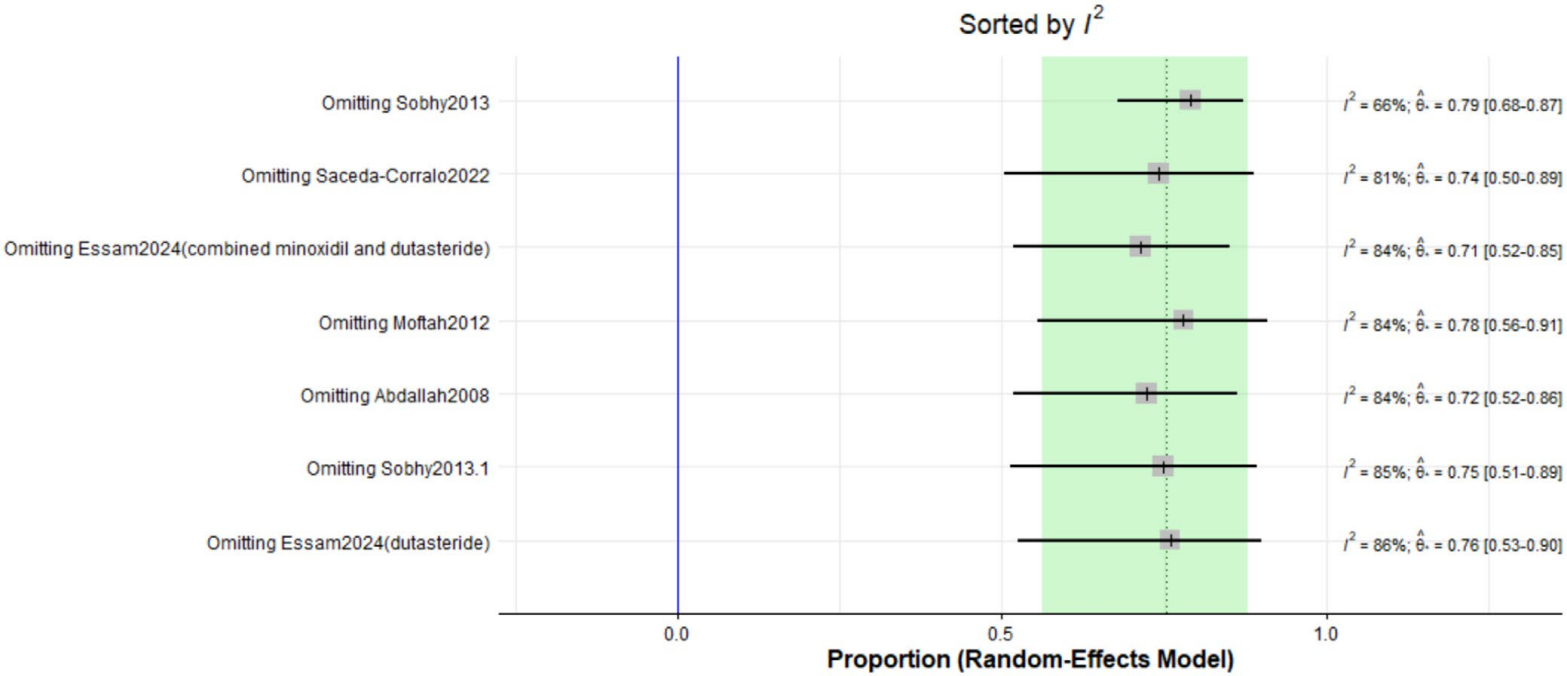
Sensitivity test of improvement rate outcome.

**FIGURE 13:**
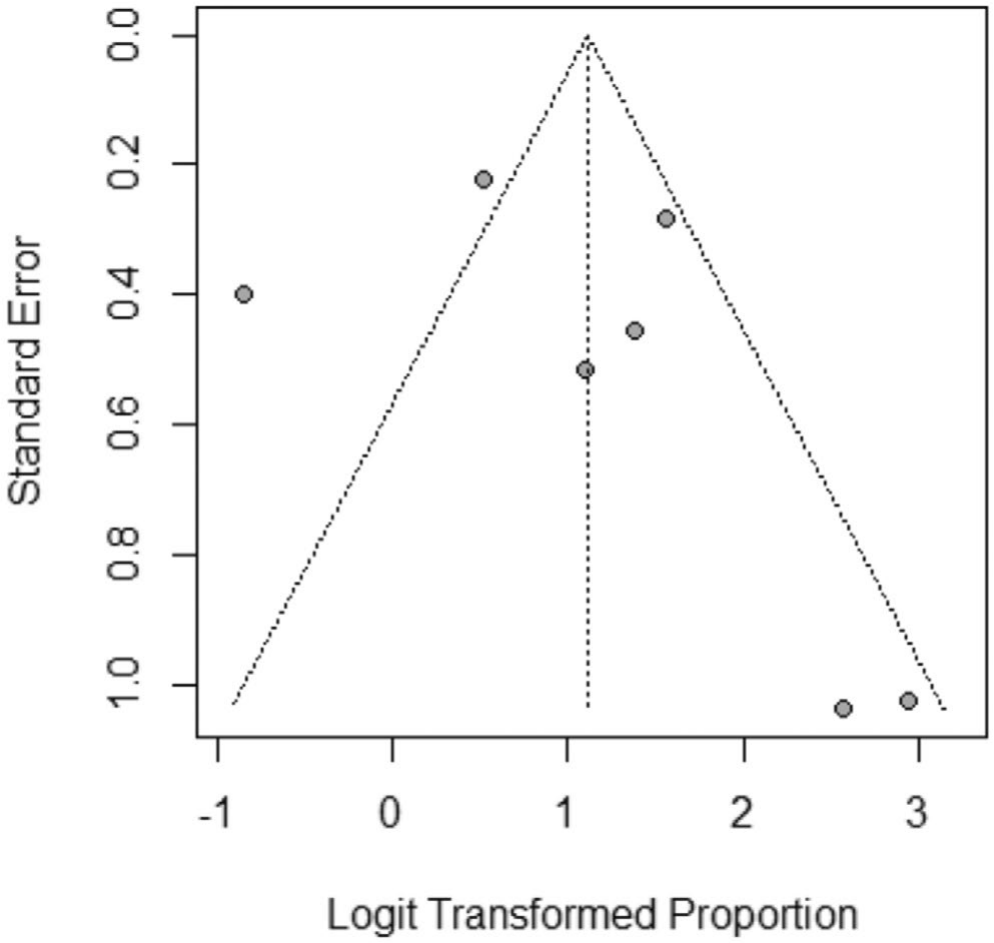
Funnel plot of improvement rate outcome.

The pooled prevalence of the improvement rate after Dutasteride was 75% with a 95% confidence interval (CI) ranging from 0.56 to 0.88 (Figure 11). The result was statistically significant (*p* < 0.0001). The sensitivity analysis revealed substantial heterogeneity among our included (*I* ^2^ = 82.8%). The leave‐one‐out test decreased the heterogeneity after omitting Sobhy 2013 (*I* ^2^ = 66%) (Figure 12). The funnel plot of improvement rate is shown in (Figure 13).

##### 3.2.4.2 Drug Subgrouping

**FIGURE 14:**
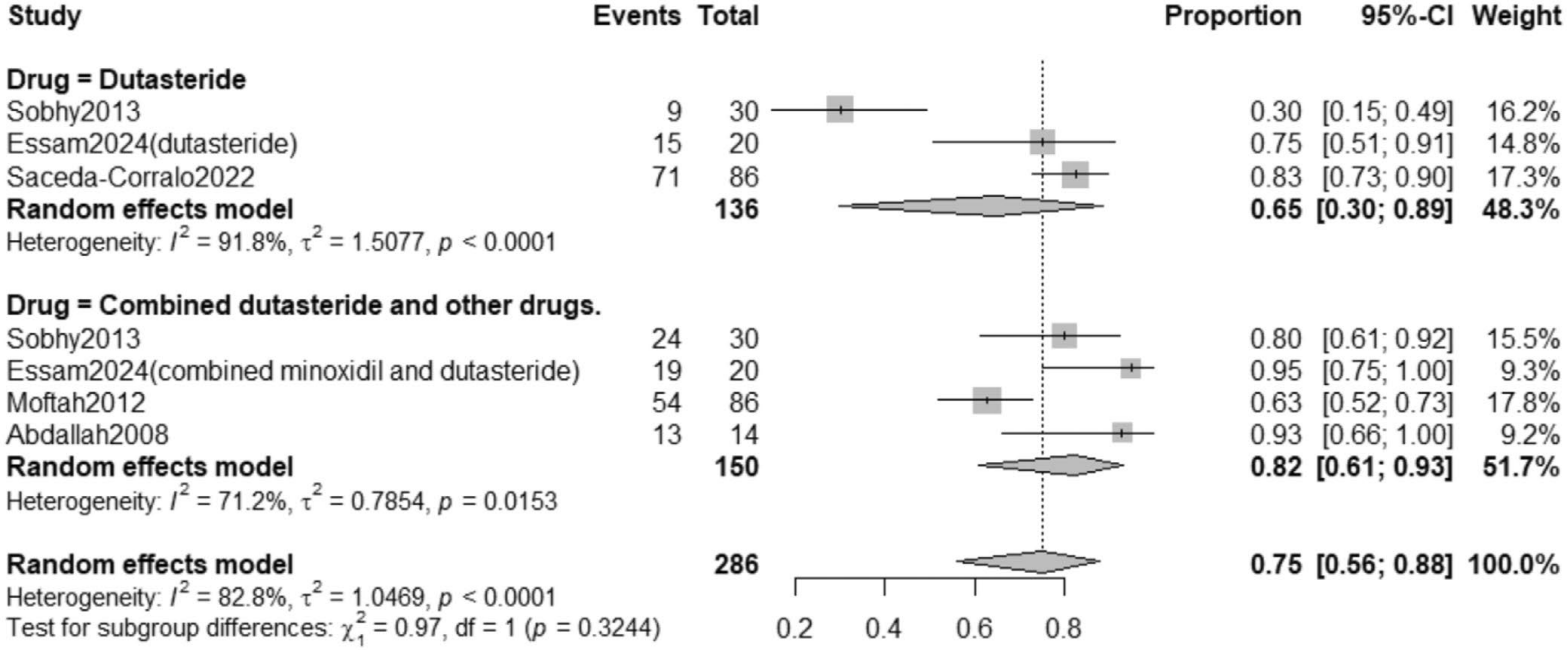
Subgrouping of improvement rate outcome based on drug.

The subgroup analysis revealed that the pooled prevalence of improvement after Dutasteride only was 65% with a 95% confidence interval (CI) ranging from 0.30 to 0.89 with a statistically significant (*p* < 0.0001) result. In contrast, the pooled prevalence of improvement after Combined Dutasteride and other drugs was 82% with a 95% confidence interval (CI) ranging from 0.61 to 0.93 with a statistically significant (*p* = 0.0153) result (Figure 14).

##### 3.2.4.3 Duration Subgrouping

**FIGURE 15:**
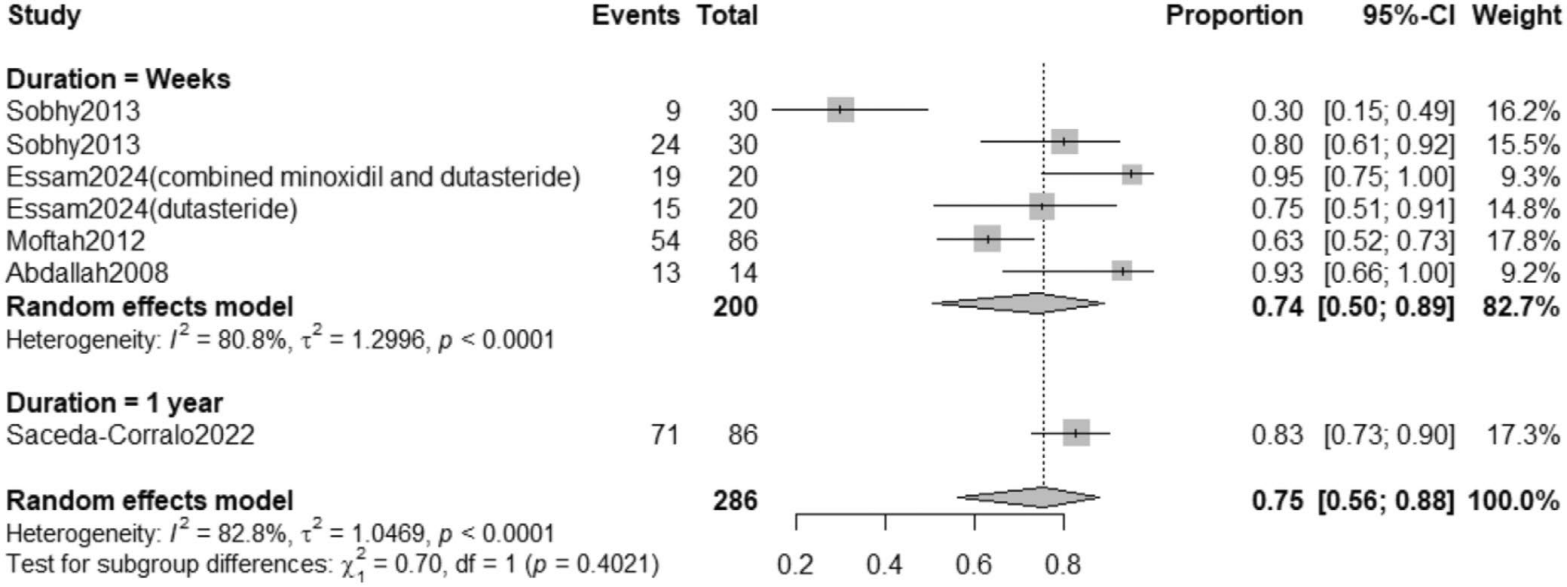
Subgrouping of improvement rate outcome based on duration.

The subgroup analysis revealed that the pooled prevalence of improvement rate after treatment for weeks was 74% with a 95% confidence interval (CI) ranging from 0.50 to 0.89 with a statistically significant (*p* < 0.0001) result (Figure 15).

#### 3.2.5 Total Adverse Effects

##### 3.2.5.1 Primary Analysis

**FIGURE 16:**
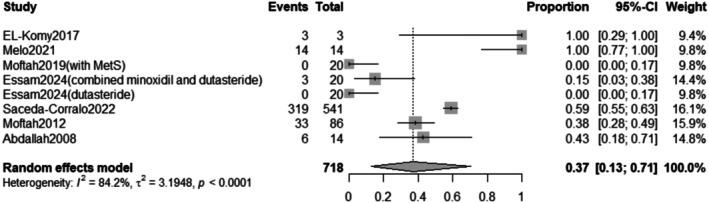
Forest plot of total adverse effects outcome.

**FIGURE 17:**
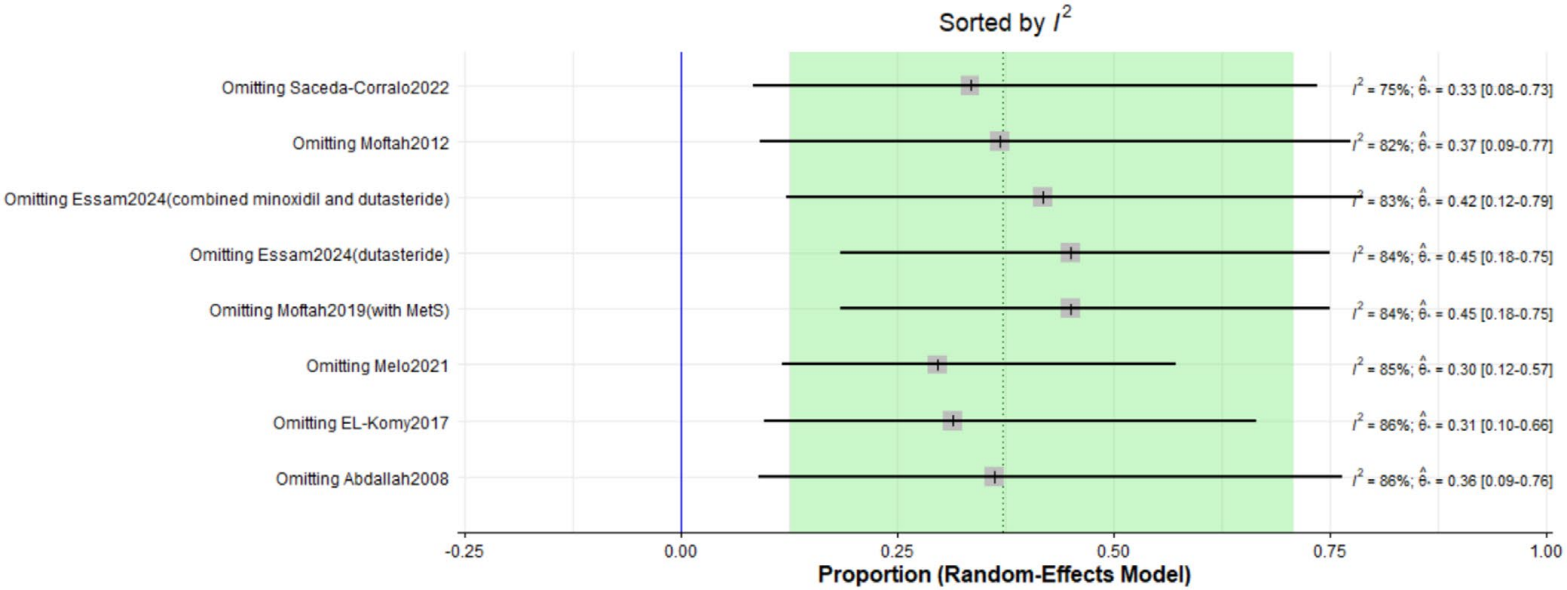
Sensitivity test of total adverse effects outcome.

**FIGURE 18:**
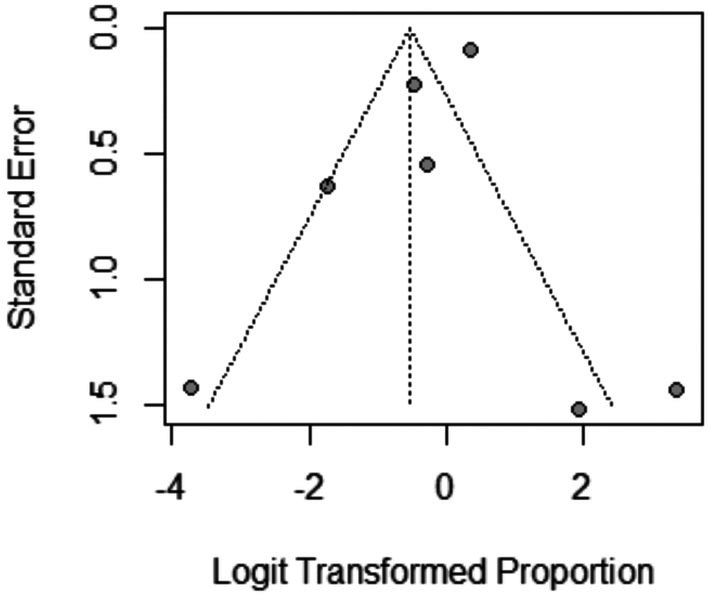
Funnel plot of total adverse effects outcome.

The pooled prevalence of the total adverse effects after Dutasteride was 37% with a 95% confidence interval (CI) ranging from 0.13 to 0.71 (Figure 16). The result was statistically significant (*p* < 0.0001). The sensitivity analysis revealed substantial heterogeneity among our included (*I* ^2^ = 84.2%). The leave‐one‐out test did not resolve the heterogeneity (Figure 17). The funnel plot of total adverse effects is shown in (Figure 18).

##### 3.2.5.2 Drug Subgrouping

**FIGURE 19:**
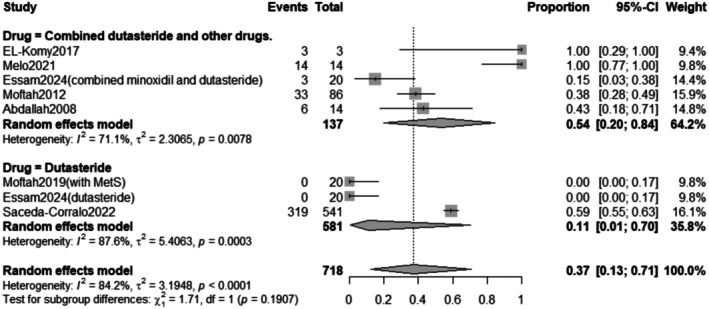
Subgrouping of total adverse effects outcome based on drug.

The subgroup analysis revealed that the pooled prevalence of total adverse effects after combined Dutasteride and other drugs was 54% with a 95% confidence interval (CI) ranging from 0.20 to 0.84 with a statistically significant (*p* = 0.0078) result. However, the prevalence of total adverse effects after Dutasteride only was 11% with a 95% confidence interval (CI) ranging from 0.01 to 0.70 with a statistically significant (*p* = 0.0003) result (Figure 19).

##### 3.2.5.3 Duration Subgrouping

**FIGURE 20:**
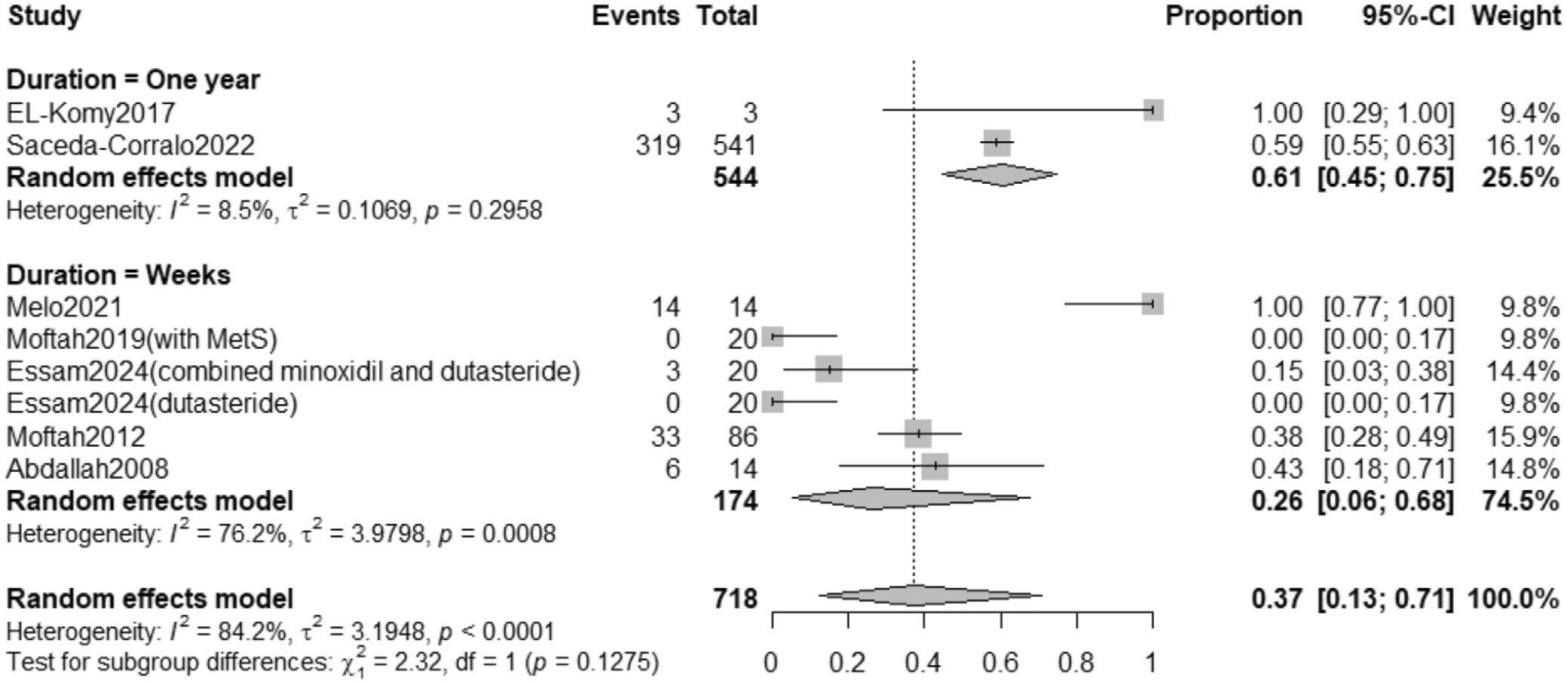
Subgrouping of total adverse effects outcome based on duration.

The subgroup analysis revealed that the pooled prevalence of total adverse effects after treatment for 1 year was 61% with a 95% confidence interval (CI) ranging from 0.45 to 0.75 with a non‐statistically significant (*p* = 0.2958) result. In contrast, the pooled prevalence of total adverse effects after treatment for weeks was 26% with a 95% confidence interval (CI) ranging from 0.06 to 0.68 with a statistically significant (*p* = 0.0008) result (Figure 20).

#### 3.2.6 Pain

##### 3.2.6.1 Primary Analysis

**FIGURE 21:**
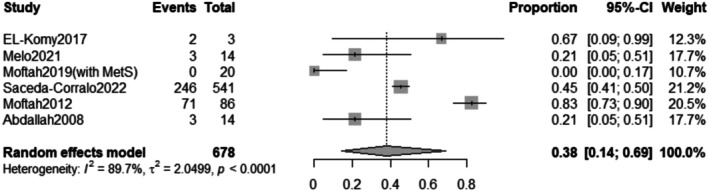
Forest plot of pain outcome.

**FIGURE 22:**
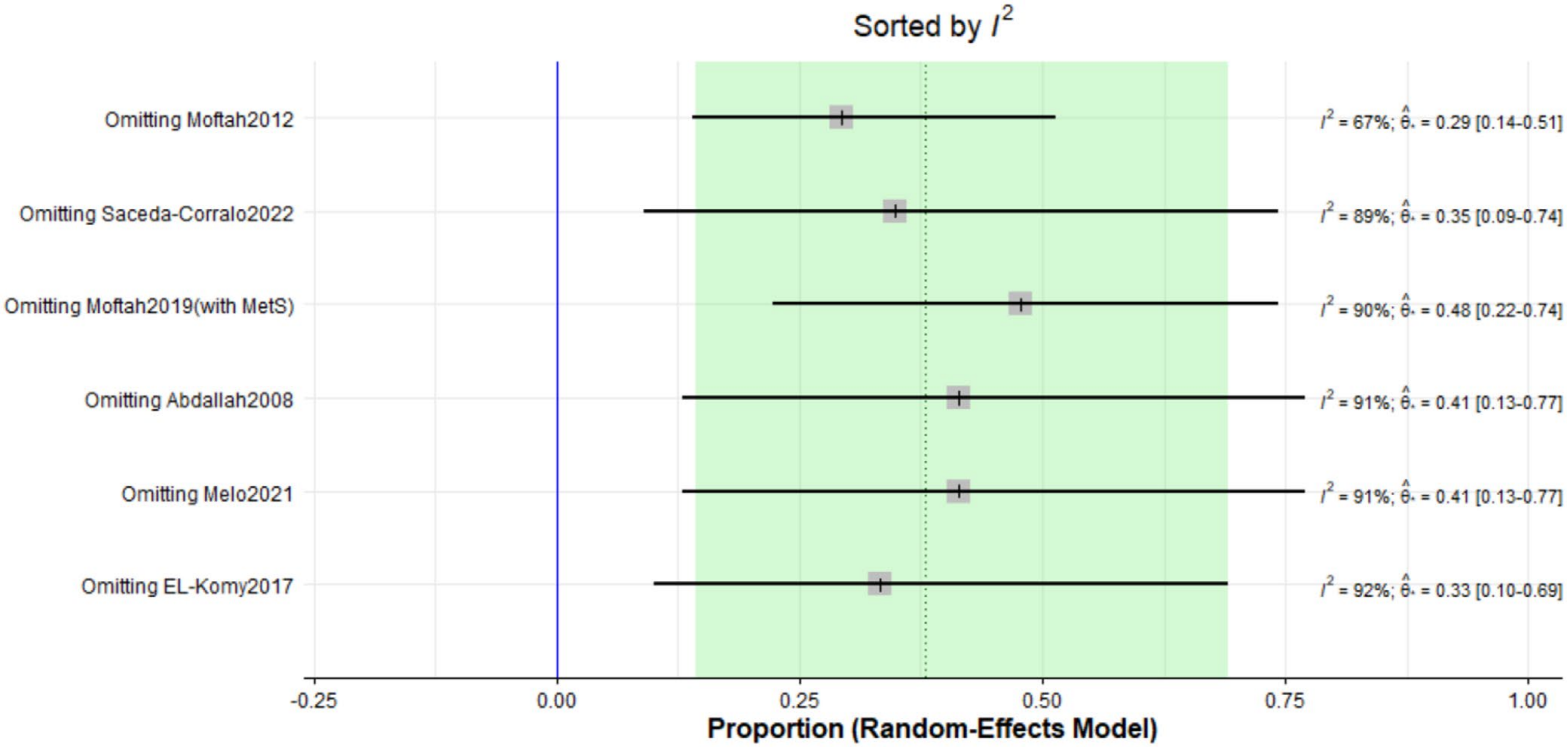
Sensitivity test of pain outcome.

**FIGURE 23:**
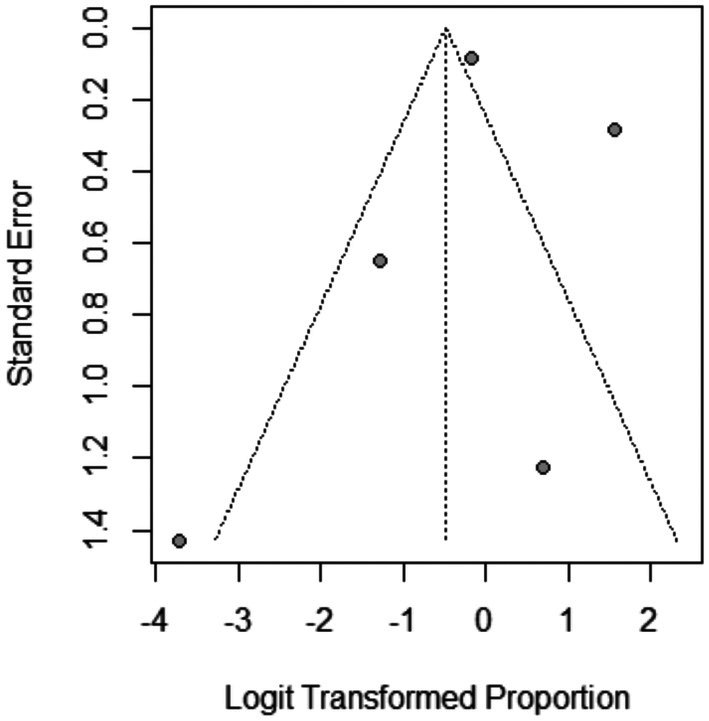
Funnel plot of pain outcome.

The pooled prevalence of the pain after Dutasteride was 38% with a 95% confidence interval (CI) ranging from 0.14 to 0.69 (Figure 21). The result was statistically significant (*p* < 0.0001). The sensitivity analysis revealed substantial heterogeneity among our included studies (*I* ^2^ = 89.7%). The leave‐one‐out test decreased the heterogeneity after omitting Moftah (*I* ^2^ = 67%) (Figure 22). The funnel plot of pain is shown in (Figure 23).

##### 3.2.6.2 Drug Subgrouping

**FIGURE 24:**
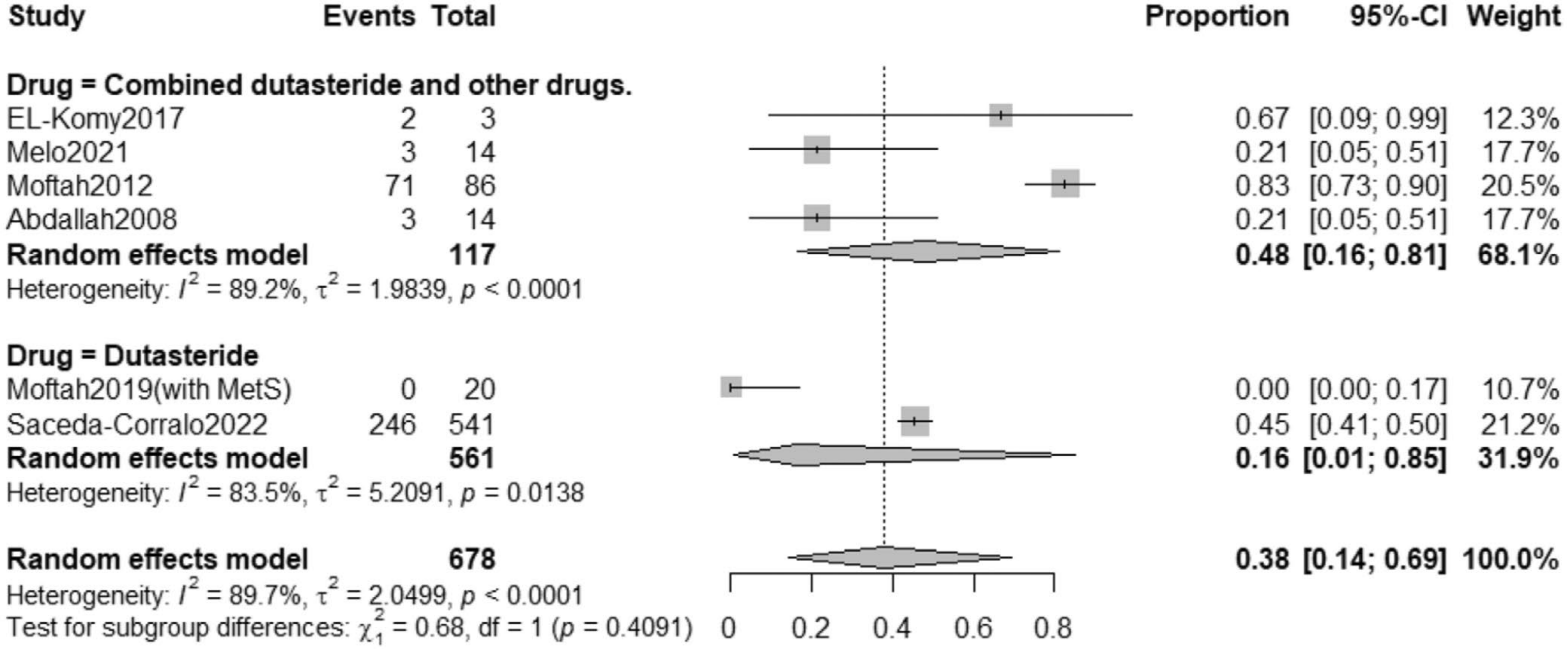
Subgrouping of pain outcome based on drug.

The subgroup analysis revealed that the pooled prevalence of pain effects after combined Dutasteride and other drugs was 48% with a 95% confidence interval (CI) ranging from 0.16 to 0.81 with a statistically significant (*p* < 0.0001) result. However, the prevalence of pain after Dutasteride only was 16% with a 95% confidence interval (CI) ranging from 0.01 to 0.85 with a statistically significant (*p* = 0.0138) result (Figure 24).

##### 3.2.6.3 Duration Subgrouping

**FIGURE 25:**
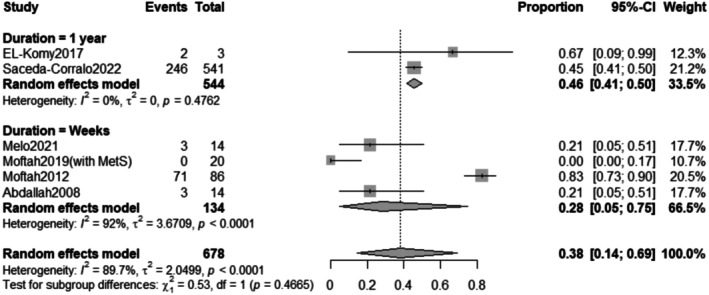
Subgrouping of pain outcome based on duration.

The subgroup analysis revealed that the pooled prevalence of pain after treatment for 1 year was 46% with a 95% confidence interval (CI) ranging from 0.41 to 0.50 with a non‐statistically significant (*p* = 0.4762) result. In contrast, the pooled prevalence of pain after treatment for weeks was 28% with a 95% confidence interval (CI) ranging from 0.05 to 0.75 with a statistically significant (*p* < 0.0001) result (Figure 25).

### 3.3 Risk of Bias Assessment

Results of the risk of bias evaluation of the included studies are shown in Table 2. A methodological quality assessment of the included non‐randomized studies was conducted using the Methodological Index for Non‐Randomized Studies (MINORS) [25]. Four of MINORS's 12 components are unique to comparative research, whereas the first eight are applicable to all study designs. The ratings for each criterion range from 0 to 2, where 0 denotes no reporting, 1 denotes insufficient reporting, and 2 denotes sufficient reporting. Comparative studies can receive up to 24 points, but non‐comparative research can only score up to 16 points.

**TABLE 2:** The Risk of Bias Assessment using the Methodological Index for Non‐Randomized Studies (MINORS) assessment tool.

Study ID	Study design	(1) A clearly stated aim	(2) Inclusion of consecutive patients	(3) Prospective collection of data	(4) Endpoints appropriate to the aim of the study	(5) Unbiased assessment of the study endpoint	(6) Follow‐up period appropriate to the aim of the study	(7) Loss to follow‐up less than 5%	(8) Prospective calculation of the study size	(9) An adequate control group (comparative studies only)	(10) Contemporary groups (comparative studies only)	(11) Baseline equivalence of groups (comparative studies only)	(12) Adequate statistical analyses (comparative studies only)	Total score	Risk of bias
Saceda Corralo et al. (2022) [23]	Retrospective study	2	2	1	2	1	1	0	0	Non‐comparative study				9/16	Moderate Risk
Melo et al. (2022) [18]	Multicenter retrospective, descriptive study	2	2	1	2	1	1	0	0	Non‐comparative study				9/16	Moderate Risk
El‐Komy et al. (2017) [24]	Case series	2	2	0	2	1	1	0	0	Non‐comparative study				8/16	moderate Risk
Moftah et al. (2013) [16]	Prospective cohort study	2	2	2	2	2	1	0	1	Non‐comparative study				12/16	Low Risk
Essam et al. (2024) [15]	Prospective comparative study	2	2	2	2	2	2	0	1	2	1	1	2	19/24	Moderate Risk

*Note:* Score each item on a scale of 0 to 2, whereby; 0: Not reported; 1: Reported but inadequate; 2: Reported and adequate.

Two separate reviewers assessed each study, and discrepancies in scores were settled by discussion or by seeking input from a third reviewer. There was a variation in the methodological rigor of the investigations, as seen by the MINORS scores, which ranged from 8 to 12 out of 16 for non‐comparative studies and from 19 to 22 out of 24 for comparative studies. The research conducted by Saceda Corralo et al., Melo et al., and EL‐Komy et al. received scores of 9/16, 9/16, and 8/16, respectively, indicating a moderate risk of bias. Furthermore, a study by Essam et al. had a score of 19/24, indicating a moderate risk of bias due to methodological flaws. This shows that the included studies' methodological quality is generally moderate. Nevertheless, items 7 (loss to follow‐up fewer than 5%) and 8 (prospective estimation of the sample size) had the most commonly found methodological flaws. Because the methodological quality of the included studies was generally moderate, the study's findings should be interpreted cautiously.

### 3.4 Cochrane Risk of Bias Assessment

**FIGURE 26:**
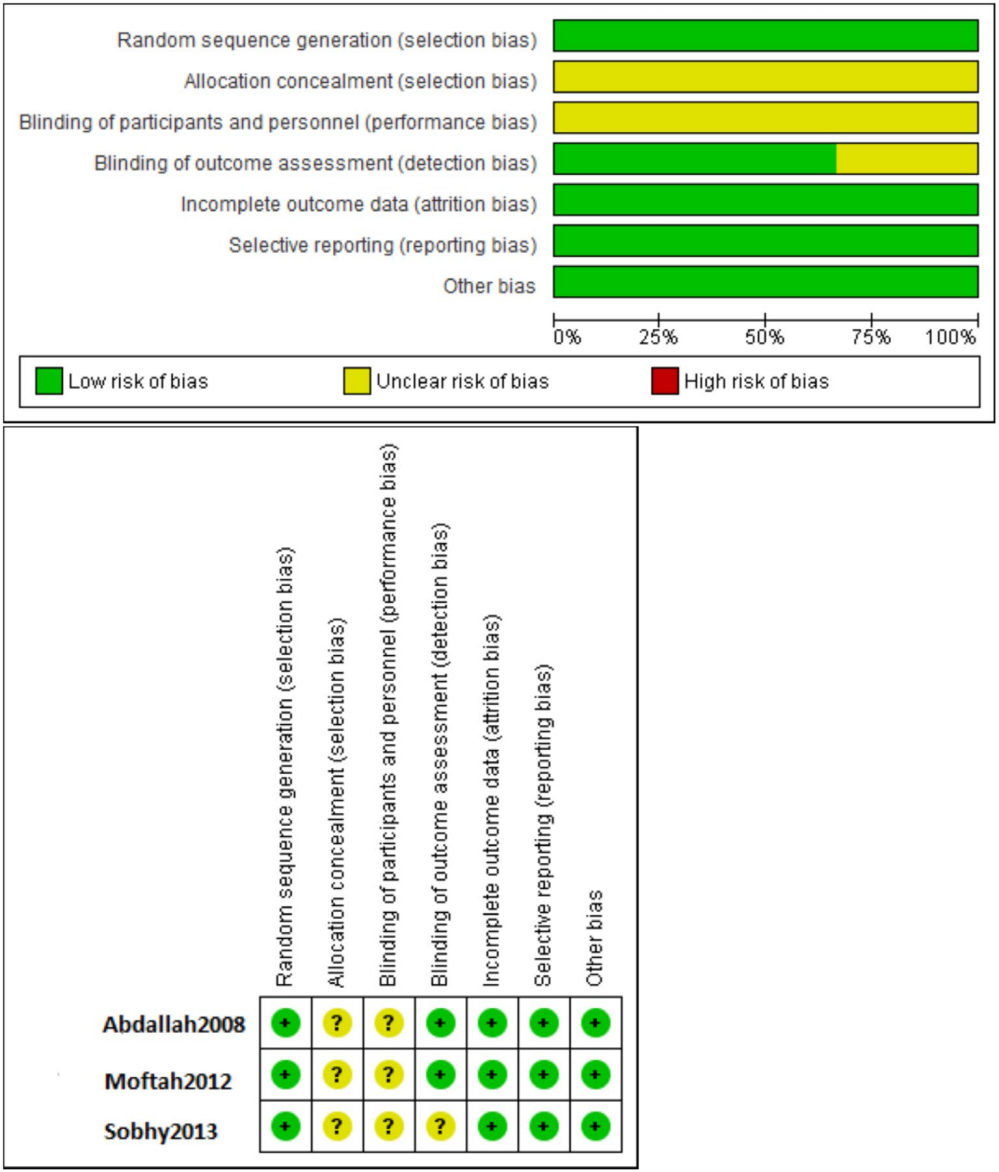
A summary of studies' risk of bias of each item.

In a number of items, the included three RCTs had a low risk of bias, especially, in random sequence generation, attrition, reporting and other form biases. However, blinding of the participants, allocation concealmentand blinding of outcome showed to have an unclear risk of bias. Addressing this issue in the discussion of the findings would increase the reliability of the results. Generally, 4/7 items were of excellent quality indicating the RCTs included were of good quality (see Figure 26).

## 4 Discussion

This systematic review and meta‐analysis consolidates evidence on the effectiveness and safety of intralesional dutasteride for treating androgenic alopecia (AGA). The results suggest that intralesional dutasteride is a promising treatment, though the current evidence has notable limitations.

Our meta‐analysis of eight studies indicates that intralesional dutasteride improves hair growth outcomes. The pooled analysis for the change in terminal hair count showed a statistically significant mean difference of 8.73 (95% CI: −4.53 to 21.99, *p* < 0.0001). However, this result showed substantial heterogeneity (*I* ^2^ = 96.3%). Subgroup analysis revealed that when dutasteride was combined with other drugs, the effect was more pronounced (mean difference = 24.25) compared to dutasteride alone (mean difference = 3.57). Similarly, the treatment led to a significant decrease in vellus hair count, with a pooled mean difference of −6.04 (95% CI: −21.80 to 9.72, *p* < 0.0001), also with high heterogeneity (*I* ^2^ = 93.4%). In contrast, the analysis for hair density did not show a significant improvement, with a pooled mean difference of −1.37 (95% CI: −16.89 to 14.15).

The overall improvement rate after treatment was 75% (95% CI: 0.56 to 0.88, *p* < 0.0001). Subgroup analysis indicated a higher improvement rate when dutasteride was combined with other drugs (82%) versus dutasteride alone (65%). The duration of treatment did not show a statistically significant difference in improvement rates between treatment for weeks (74%) and for 1 year (83%). These findings align with previous studies suggesting the potential synergistic effects of combination therapies.

The safety profile of intralesional dutasteride appears manageable. The pooled prevalence of total adverse effects was 37% (95% CI: 0.13 to 0.71). Subgroup analysis showed a lower incidence of adverse effects with dutasteride monotherapy (11%) compared to combination therapy (54%). Shorter treatment durations (weeks) were associated with a lower prevalence of adverse effects (26%) compared to longer durations (1 year, 61%). Pain was a common adverse effect, with a pooled prevalence of 38% (95% CI: 0.14 to 0.69). Similar to total adverse effects, pain was less frequent in the dutasteride‐only subgroup (16%) compared to the combined therapy group (48%). Importantly, systemic side effects commonly associated with oral dutasteride, such as sexual dysfunction, were not reported in the included studies, supporting the localized safety of this administration route.

The methodological quality of the included studies varied. The risk of bias for non‐randomized studies was assessed as moderate, with MINORS scores ranging from 8 to 12 for non‐comparative studies and 19 for one comparative study. Common methodological issues included the lack of prospective sample size calculation and high loss to follow‐up. For the three included randomized controlled trials, the risk of bias was generally low, though there was an unclear risk related to blinding of participants and allocation concealment. This variability, along with small sample sizes and differing treatment protocols, contributes to the high heterogeneity observed in our meta‐analysis and limits the strength of the conclusions.

The findings of this review are consistent with another recent research study. For example, Herz‐Ruelas et al. (2020) [21] found both oral and intralesional dutasteride to be effective, though oral administration showed a greater increase in hair count. This underscores the need for direct, head‐to‐head comparative studies. Our results also align with the broader literature on 5‐alpha reductase inhibitors, which confirm their efficacy while highlighting concerns about side effects. The absence of systemic side effects in our review of intralesional dutasteride is a significant finding that warrants further investigation.

## 5 Conclusion

This systematic review and meta‐analysis supports the use of intralesional dutasteride as an effective and relatively safe treatment for androgenic alopecia. The findings suggest that combination therapy may yield higher improvement rates but also a greater incidence of local adverse effects like pain. While systemic side effects were notably absent, the evidence is constrained by the methodological limitations of the included studies, including heterogeneity in protocols and small sample sizes. To establish definitive clinical recommendations, future research should prioritize large‐scale, multicenter randomized controlled trials with standardized methodologies, objective outcome measures, and long‐term follow‐up.

## Author Contributions

Abdullah Almeziny, A.I.A. and S.A. designed the research study, conducted the literature search, and drafted the manuscript. A.G. and A.L. assisted in the literature search and data extraction. N.M. and A.M. contributed to data analysis. A.I.A. and S.A. provided critical revisions and supervised the work. All authors have read and approved the final manuscript. Make it more formal and prepare it for a systematic review.

## Conflicts of Interest

The authors declare no conflicts of interest.

## Data Availability

The data that support the findings of this study are available from the corresponding author upon reasonable request.
